# Broad-spectrum antifungal activities and mechanism of drimane sesquiterpenoids

**DOI:** 10.15698/mic2020.06.719

**Published:** 2020-03-12

**Authors:** Edruce Edouarzin, Connor Horn, Anuja Paudyal, Cunli Zhang, Jianyu Lu, Zongbo Tong, Guri Giaever, Corey Nislow, Raja Veerapandian, Duy H. Hua, Govindsamy Vediyappan

**Affiliations:** 1 Department of Chemistry, 1212 Mid Campus Drive North, Kansas State University, Manhattan, KS 66506 USA.; 2 Division of Biology, 1717 Claflin Road, Kansas State University, Manhattan, KS 66506 USA.; 3 Faculty of Pharmaceutical Sciences, University of British Columbia, Vancouver, BC Canada V6T 1Z3.

**Keywords:** drimenol, synthesis, Candida albicans, Candida auris, Saccharomyces cerevisiae, fungicidal antifungal, novel mechanism of action, Crk1 kinase associated

## Abstract

Eight drimane sesquiterpenoids including (-)-drimenol and (+)-albicanol were synthesized from (+)-sclareolide and evaluated for their antifungal activities. Three compounds, (-)-drimenol, (+)-albicanol, and (1*R*,2*R*,4a*S*,8a*S*)-2-hydroxy-2,5,5,8a-tetramethyl-decahydronaphthalene-1-carbaldehyde (4) showed strong activity against *C. albicans*. (-)-Drimenol, the strongest inhibitor of the three, (at concentrations of 8 – 64 µg/ml, causing 100% death of various fungi), acts not only against *C. albicans* in a fungicidal manner, but also inhibits other fungi such as *Aspergillus, Cryptococcus, Pneumocystis, Blastomyces, Saksenaea* and fluconazole resistant strains of *C. albicans, C. glabrata, C. krusei, C. parapsilosis* and *C. auris.* These observations suggest that drimenol is a broad-spectrum antifungal agent. At a high concentration (100 μg/ml) drimenol caused rupture of the fungal cell wall/membrane. In a nematode model of *C. albicans* infection, drimenol rescued the worms from *C. albicans*-mediated death, indicating drimenol is tolerable and bioactive in metazoans. Genome-wide fitness profiling assays of both *S. cerevisiae* (nonessential homozygous and essential heterozygous) and *C. albicans* (Tn-insertion mutants) collections revealed putative genes and pathways affected by drimenol. Using a *C. albicans* mutant spot assay, the Crk1 kinase associated gene products, Ret2, Cdc37, and orf19.759, orf19.1672, and orf19.4382 were revealed to be involved in drimenol's mechanism of action. The three orfs identified in this study are novel and appear to be linked with Crk1 function. Further, computational modeling results suggest possible modifications of the structure of drimenol, including the A ring, for improving the antifungal activity.

## INTRODUCTION

Fungi have emerged in the last two decades as major causes of human disease. *Candida albicans* is a major fungal pathogen affecting humans of all ages and is the fourth leading cause of nosocomial bloodstream infections in the US [1]. *C. albicans* is the most frequently found fungal pathogen in humans and costs the US health care system around $3 billion annually due to treatment costs and lost productivity [2, 3]. According to a recent report the total global costs due to productivity loss caused by Candidiasis in women was estimated to be over $14 billion in 2010 [4]. *C. albicans*, a polymorphic fungus, exists as yeast, pseudohyphal and hyphal forms, with each contributing to its virulence. While the yeast form is essential for dissemination, the hyphal form is critical for invasion of cells, immune evasion, and biofilm formation. Furthermore, the ability to switch between forms is also essential for pathogenicity.

*C. albicans* and other *Candida* spp. cause mucosal and disseminate invasive candidiasis, especially among patients who are immunocompromised or hospitalized with serious underlying diseases. The overall mortality of invasive diseases caused by *Candida* spp. and *Aspergillus* spp. is around 50% [1, 5]. While there are more than 150 species of *Candida*, about 15 species are recognized as frequent human pathogens [5, 6]. Some of them are: *C. albicans, C. glabrata, C. krusei, C. tropicalis* and *C. parapsilosis*. Among these, *C. albicans* is by far the most common species isolated from humans and is a frequent denizen of the oropharynx, mucousal surfaces, gastrointestinal and genitourinary tracts. *C. auris*, an emerging *Candida* strain, was first discovered in 2009 in Southeast Asia and is now present in 33 countries across six continents. The mortality rate of *C. auris* infection is high since it is resistant to almost all antifungals available, it can grow invasively and causes skin infections [7].

In the developing world, there are ∼1 million cases of cryptococcal diseases per year resulting in 675,000 deaths [8, 9]. *Cryoptococcus neoformans* is an opportunistic fungal pathogen that causes meningitis in immunocompromised individuals. Often found in soils contaminated with bird feces, *C. neoformans* enters its host through the lungs via inhalation of spores. Some of the cryptococcal species are hypervirulent [10] and have drawn a considerable public attention due to their causative role in the cryptococcosis outbreak throughout the Pacific Northwest [11, 12]. Only few antifungals are useful to treat cryptococcosis and drug resistant strains are emerging.

*Aspergillus* spp. are ubiquitous molds found widely in the environment as saprophytes and produce microscopic spores or conidia which, upon inhalation, cause invasive pulmonary disease. In immunocompromised patients having hematopoietic stem cell transplantation, solid organ transplantation, or chemotherapy, invasive aspergillosis remains the major cause of infection-related mortality [13, 14]. Among several species of *Aspergillus, A. fumigatus* and *A. flavus* are common pathogens.

Dermatophytes are another group of keratinophilic pathogenic fungi that cause a variety of infections in humans and animals [15]. Some of these fungi include *Trichophyton tonsurans* (scalp ring-worm), *T. equinum*, and *Microsporum gypseum* (garderner's ringworm). Emerging fungal diseases such as zygomycosis are life-threatening particularly during natural calamity (e.g. the 2004 tsunami, the 2008 Katrina and May 2011 Joplin tornado). Molecules with broad-spectrum antifungal activity are highly desirable to combat various fungal pathogens.

Because fungi are eukaryotes, the development of antifungal therapeutics that are nontoxic to humans is challenging due to the availability of relatively few targets. In the last twenty years, only one new class of antimycotic (β-glucan synthase inhibitor, the echinocandins) was introduced into clinical practice. Although this drug is an important addition, it has a number of limitations including ineffectiveness against *Cryptococcus* spp. and poor oral bioavailability [16]. Currently, the antifungal therapeutic options are limited, especially when compared to available antibacterial agents [2, 17–19]. Among the five classes of antifungals, azoles, echiocandins, polyenes, allylamines, and pyrimidine derivatives, only three are used clinically: azoles, echiocandins, and polyenes. Azole drugs, such as fluconazole (FLU), inhibit ergosterol synthesis through inhibition of lanosterol 14-α-demethylase, impairing formation of the fungal cell membrane. Echocandins, such as caspofungin (CAS), block 1,3-β-glucan synthase and lead to depletion of glucan in the fungal cell wall. Polyenes, including amphotericin B (AMB), bind to ergosterol in the fungal cell membrane and change the cell membrane transition temperature, resulting in the leakage of ions and small organic molecules, and eventual cell death. Allylamines, such as amorolfin, affect ergosterol synthesis by the inhibition of squalene epoxidase. Pyrimidines, such as flucytosine (or 5-fluorocytosine), block nucleic acid synthesis, leading to the impediment of protein synthesis [20, 21]. Although a new antifungal drug, isavuconazonium sulfate, belonging to azole family, has been developed in 2015 [22], no new ‘class' of antifungal agent has been approved by the Food and Drug Administration (FDA) since 2006 [18, 23, 24].. Thus, the invention of new antifungal classes to overcome the increasing emergence of antifungal drug resistance is greatly needed.

**Figure 1:**
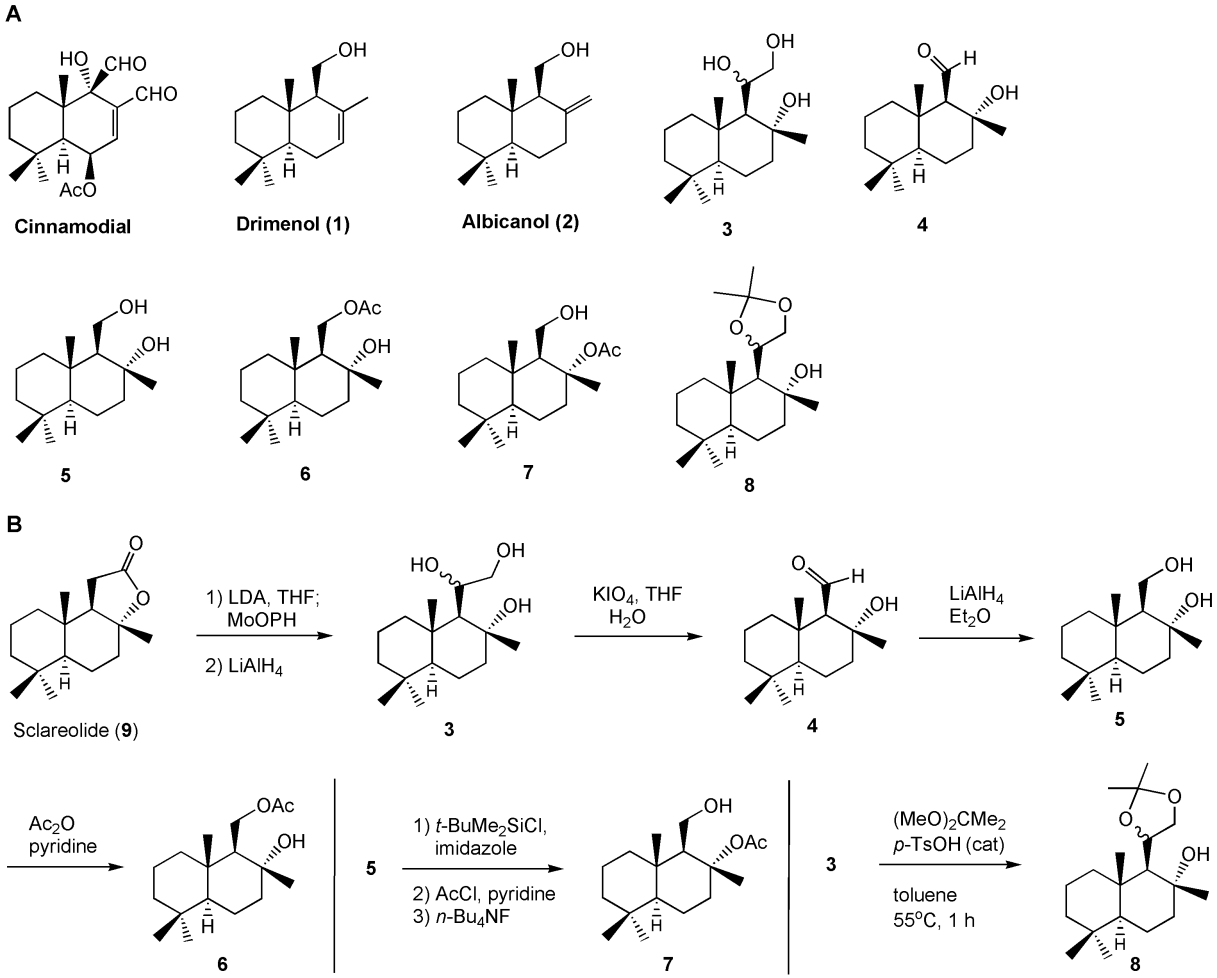
FIGURE 1: Bioevaluation of drimane sesquiterpenoids for their antifungal activities (A) and their (3 – 8) synthesis (B).

Traditional antimycotics have drawbacks, including toxicity to human cells, a limited range of cellular targets, the development of antifungal resistance [3, 9, 25, 26], and the failure to successfully control pathogenesis. To develop new antifungal agents based on drimane sesquiterpenes, we have investigated synthetic drimane terpenes, (-)-drimenol (**1**) and (+)-albicanol (**2**), along with six analogs, **3** – **8** (**Figure 1A**), for their antifungal activities and identified (-)-**1** as a potent broad-spectrum fungicidal agent. Moreover, we determined their mechanism of action through forward genetic screening of mutant libraries of *C. albicans* and baker's yeast and found that (-)-**1** affects Crk1 kinase-dependent gene products involved in protein secretion and vacuolar biogenesis in fungi.

## RESULTS

Several drimane sesquiterpenoids were synthesized in our laboratory during the total synthesis of (+)-chloropuupehenone, a natural product from marine sponges [27]. Based on the antimycobacterial activity of sesquiterpene natural product, cinnamodial, isolated from *Warburgia salutaris* (a species of tree in the Canellaceae family) [28], we anticipated that drimane sesquiterpenes and closely related compounds [29] could be effective antimycotics against *C. albicans*. Two other related antifungal compounds, muzigadial and warburganal, were also isolated from the *Warburgia ugandensis* plant [30]. Five representative drimane terpenes, **1** – **5**, along with their derivatives, **6** – **8** (**Figure 1A**), were screened for their ability to inhibit *C. albicans* growth. It was assumed that additional hydroxyl group(s) or oxygen atoms in the molecule enhances water solubility and may improve bioactivity [31]. Molecules **3** – **7** possess extra hydroxyl, aldehyde, or acetoxy functions and molecule **8** contains an acetonide moiety in the drimane structure.

Compounds **1** – **5** were prepared by following previously reported methods [27]. Molecule **6** was made from a mono-acetylation of **5** with acetic anhydride and pyridine in dichloromethane (**Figure 1B**). Molecule **7** was obtained by a sequence of three reactions: (i) silylation of the primary alcohol with *t*-butyldimethylsilyl chloride and imidazole in dichloromethane; (ii) acetylation of the tertiary alcohol with acetyl chloride and pyridine; and (iii) removal of the silyl ether group with tetra-*n*-butylammonium fluoride in THF. Compound **8** was produced from the treatment of triol **3** with 2,2-dimethoxylpropane and a catalytic amount of *p*-toluenesulfonic acid in toluene. The experimental procedures are described in the Materials and Methods section.

Examination of the antifungal activities of these molecules along with the mechanistic study of the most active molecule may allow future improvement in bioactivity and reduction in toxicity. We used the *C. albicans* strain SC5314 for our initial screening of antifungal activities of drimane sesquiterpenoids **1** – **8**. The compounds were solubilized in dimethyl sulfoxide (DMSO), 10 mg/ml, as stock solutions and stored at −20°C. Prior to assays, stock solutions of compounds were diluted to 200 – 12.5 µg/ml in the growth media for yeast antifungal assays using the Clinical and Laboratory Standards Institute (CLSI) M38-A2 method [32]. Fortuitously, we found (-)-drimenol (**1**) and (+)-albicanol (**2**) along with compound **4**, inhibit *C. albicans* SC5314 growth (**Table 1**). Among these three compounds, we identified **1** being more potent (with a MIC value ∼30 µg/ml) than other compounds (∼60 µg/ml); therefore **1** was used for further studies including mechanistic investigation.

**TABLE 1.** Antifungal activities of drimane sesquiterpenoids 1 – 8 against *C. albicans* SC5314.

Molecule	1	2	3	4	5	6	7	8
**Antifungal activity**	100%	50%	Inactive	75%	Inactive	Inactive	Inactive	Inactive

### Antifungal activities of drimenol against various pathogenic fungi

Since our initial assay with *C. albicans* confirmed the antifungal activities of **1**, we extended the susceptibility assays to other pathogenic fungi including FLU resistant *C. albicans*, various species of *Candida, Cryptococcus, Aspergillus*, and a dermatophyte fungus. The CLSI broth dilution methods of M27-A3 for yeasts and M38-A2 for filamentous fungi [32] were used to determine the susceptibility. Molecule **4** was not further investigated due to the presence of an aldehyde function, which may react with biological molecules.

Briefly, yeast cells or conidia (for filamentous fungi) were suspended in RPMI-1640 medium to a final concentration of 10^5^ cfu/ml and distributed in 96-well microplates to a total volume of 100 μl/well. Molecule **1** was added to the wells and two-fold serial dilutions were prepared. Du-plicates were used for each concentration and wells with or without DMSO served as controls. Plates were incubated without shaking at 37°C for 24 – 48 h for yeasts and 30°C for four days for filamentous fungi (*Aspergillus* and *Trichophyton* spp.). The MIC was defined as the lowest compound concentration at which no growth occurred, as determined visually and microscopically (inverted microscope). Representative images for the inhibition of fungi are shown in Supplementary Figure 1. **Table 2** summarizes the MIC values of **1** and positive antifungal controls including FLU, posaconazole, and voriconazole, along with additional fungi such as *C. glabrata* [BG2], *C. albicans* [FLU resistant], *C. auris,* and *T. equinum*.

**TABLE 2.** Drimenol (1) activities (in µg/ml) against various pathogenic fungi.

	**Antifungal**	**Drimenol**	**Fluconazole**	**Posaconazole**	**Voriconazole**
**Species**	**Isolate**	**No.**	**50%**	**100%**	**50%**	**100%**	**100%**
*C. parapsilosis*	CLSI QC	32	32	1	-	-
*C. krusei*	CLSI QC	32	>64	16	-	-
*P. variotii*	CLSI QC	16	32	-	≤0.03	0.125
*C. albicans*	CA1	32	32	0.125	-	-
	CA2	32	>64	0.25	-	-
	CA3	32	32	>64	-	-
*C. neoformans*	CN1	16	32	4	-	-
	CN2	8	64	64	-	-
	CN3	16	32	64	-	-
*A. fumigatus*	AF1	16	32	-	-	0.5
	AF2	8	32	-	-	2
	AF3	16	32	-	-	4
*Fusarium*	FO1	>64	>64	-	-	4
	FO2	>64	>64	-	-	8
	FS1	32	64	-	-	8
*Scedosporium*	LP1	16	>64	-	-	>16
	SA1	16	>64	-	-	1
	SB1	16	>64	-	-	2
*Rhizopus*	RA1	32	32	-	0.25	-
	RA2	32	32	-	0.25	-
	RA3	64	>64	-	0.25	-
*Apophysomyces*	AP01	32	>64	-	≤0.03	-
	AP02	32	>64	-	0.25	-
	*Saksenaea*	SAK1	16	16	-	≤0.03	-
SAK2		32	64	-	0.06	-
SAK3		4	32	-	≤0.03	-
*Blastomyces*	BD1	8	8	-	-	0.5
	BD2	4	16	-	-	0.06
	BD3	4	8	-	-	≤0.03
*C. glabrata*	BG2	30	50	-	-	-
*C. albicans*	95-98-flu resistant	30	50	-	-	-
*C. auris*	30	50	-	-	-
*Trichophyton equinum*	-	15	-	-	-

The MIC (100% growth inhibition) of **1** ranges from 4 µg/ml to 64 µg/ml. Fluconazole, posaconazole, and voriconazole (in µg/ml) were used as controls.

Results summarized in Supplementary Figure 1 show that **1** has a broad-spectrum fungicidal activity against various fungi including FLU resistant *C. albicans* and *Cryptococcus* spp. albeit, at a higher MIC concentration (50 μg/ml). However, for a dermatophyte fungus, the MIC value was lower (15 μg/ml). When compared to DMSO controls, fungi exposed to **1** showed an absence of growth. At higher concentration of **1** (100 μg/ml), *C. albicans* yeast cells lysed and released their cellular contents (Supplementary Figure 2, arrow). Consistent with this observation, **1** inhibited the germination of *A. nidulans* spores and appeared to cause swelling of germinating spores (Supplementary Figure 2, lower right).

To extend our antifungal screening with **1** against additional human pathogenic fungi, we have used the non-clinical and pre-clinical service program offered by the NIH NIAID supported fungus testing center at the University of Texas Health Sciences Center, San Antonio. The fungus testing center used the CLSI M38-A2 method [32] to determine the MIC of **1** after incubation for 24 - 72 hours with concentrations ranging from 0.125 - 64 μg/ml. Positive control antifungals (FLU, posaconazole and voriconazole) were also included in parallel. Results are summarized in **Table 2**. While the MIC for many fungi ranged from 4 – 64 μg/ml, for some fungi (*Fusarium, Scedosporium,* and *Apophysomyces*) it was above the highest concentration used (64 μg/ml). The antifungal susceptibility of these fungi to clinical antifungals (e.g. FLU) could be less.

Next, we determined the viability of fungal cells that were exposed to **1**. Cells exposed to 50 μg/ml (MIC) for 24 h or at 100 μg/ml for 48 h were used. To determine the viability of treated fungal cells, small volumes (1 - 5 μg/ml) of mixed cell suspensions were removed from wells and spotted on YPD agar medium. The agar plates were incubated at 30°C for 24 h - 72 h and the growth of fungi was recorded. Growth of yeasts occurred within 24 h and of filamentous fungi within 48 - 72 h for control (without **1**), but not for those treated with **1** suggesting that **1** acts as a fungicidal compound (data not shown).

### Drimenol acts better than FLU against *C. auris* growth

**Figure 2:**
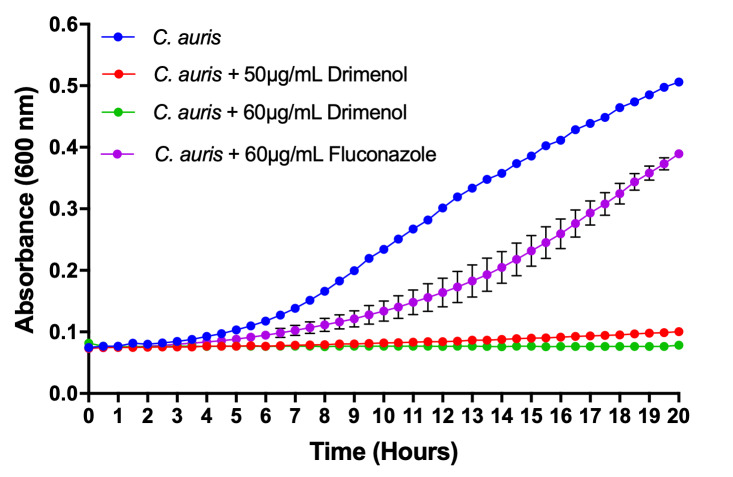
FIGURE 2: Drimenol (1) inhibits *C. auris* growth better than FLU. *C. auris* was grown in honeycomb microtiter wells containing RPMI medium in the presence and absence of **1** for 20 h at 37°C. Fungal growth was measured by absorbance at OD 600 nm using a Bioscreen-C growth monitor. Growth curves show the mean of triplicates and experiments were repeated at least twice. Error bars are SD and were too short to appear in the line graphs.

*C. auris* is an emerging multidrug resistant fungal pathogen that is known to cause nosocomial infections with “superbug”-like traits [33]. Recently, the Center for Disease Control and prevention has issued a clinical health emergency warning about this fungus. Since **1** showed a broad-spectrum fungicidal activity, we determined its effect against *C. auris* growth using a bioscreen-C growth monitoring system. *C. auris* was grown in the presence or absence of **1** in RPMI medium (CLSI method) [32] for 24 h at 37°C. Negative controls (solvent) and positive controls (FLU) were included in parallel. Results depicted in **Figure 2** indicate that **1** inhibits *C. auris* growth completely at 60 μg/ml. In contrast, FLU at the same concentration (60 μg/ml) showed poor inhibition of growth. Thus, **1** could be useful as a broad-spectrum fungicidal compound.

### Drimenol (1) is tolerated by *Caenorhabditis elegans* and protects it from fungal-mediated death

**Figure 3:**
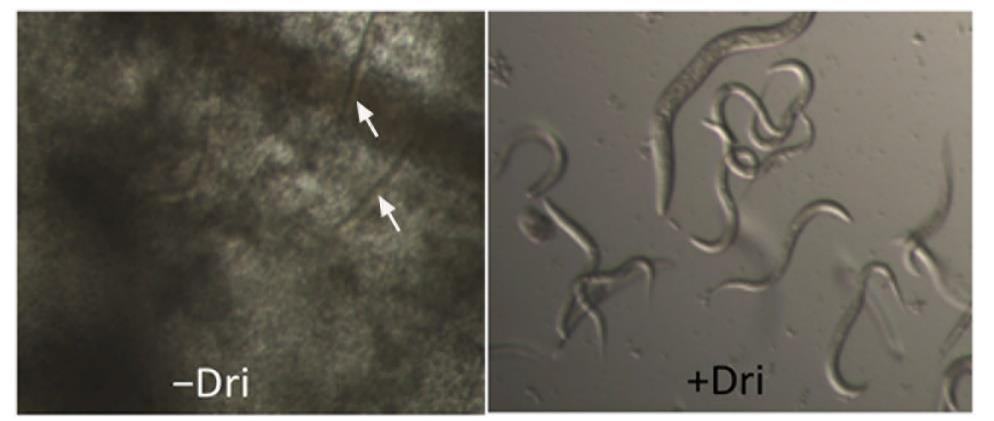
FIGURE 3: Protection of *Caenorhabditis elegans* worms from *C. albicans-*mediated death by drimenol (1). *C. albicans* (yeast cells) fed larvae were incubated in RPMI medium without and with **1** (+Dri) (50 µg/ml) in a 96 well microtiter plate and incubated at 30°C for two to three days. Left panel without **1** shows dead worms (straight and immobile, arrows) due to *C. albicans* growth. Right panel shows a well containing **1** where worms were alive as judged by their movements and the lack of fungal growth.

Invertebrate animal models provide an inexpensive and powerful platform to test antifungal compounds for their efficacy and toxicity simultaneously. We evaluated **1** for its antifungal activity and tolerance in a *C. elegans* infection model of candidiasis as described previously [34]. Results shown in **Figure 3** indicate that **1** can protect worms from *C. albicans*-mediated death and that the worms were not adversely affected by **1**, as judged by their motility and viability following compound exposure. To test the toxicity of **1** against the nematode, they were incubated with different concentrations of **1** (100, 50, 25, 12, and 6 μg/ml) for 24 h in M9 medium. **1** affected the worms very little (∼6% death and ∼94% survived) at the maximum concentration used (100 μg/ml).

### Mechanism of drimenol (1) antifungal activity

To understand the compound's mechanisms of action (MOA), researchers have used pooled libraries of genome-wide barcoded mutant collections of *Saccharomyces cerevisiae* or *C. albicans* for drug-induced sensitivity assay or the haploinsufficiency (HIP) assay [35–37]. For example, if **1** can inactivate partially or completely its protein target in the heterozygous mutant pool, the resulting growth defect of that mutant(s) can be measured quantitatively by hybridization or sequencing the tagged unique barcodes. This approach will help narrow down the putative target(s). Similarly, a homozygous nonessential mutant library can be used as a complementary approach to the heterozygous essential mutant collection to verify the target pathway/genes of compounds. In this case, if the homozygous mutant of a gene is sensitive to the compound, then that gene may not be the drug target [35] as the homozygous mutant lacks the gene product. This implies that the compound may exert its effect via drug-induced synthetic lethality. Thus, by combining data from both heterozygote and homozygote screens one may determine the compound's MOA.

**Figure 4:**
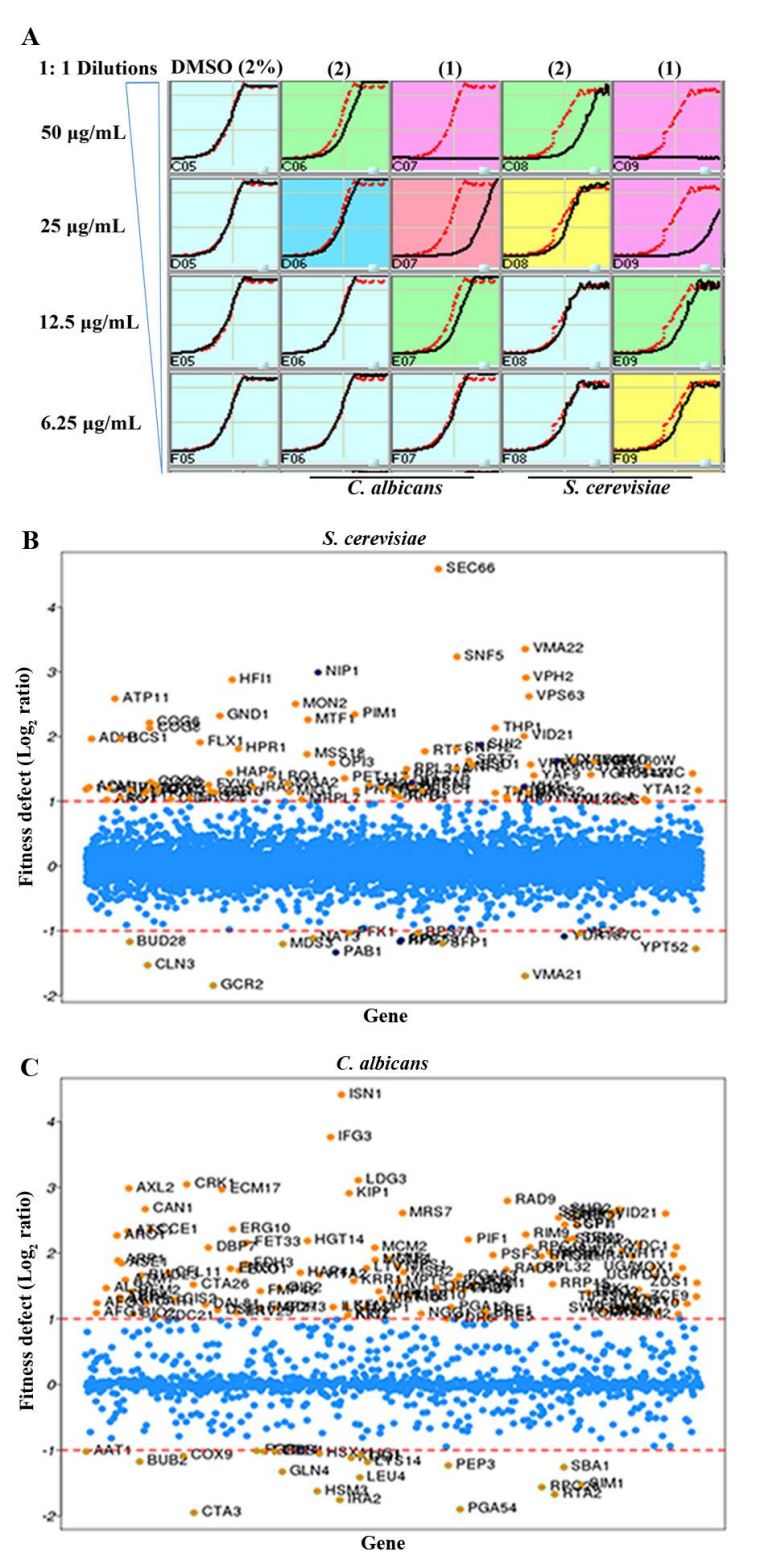
FIGURE 4: Genome-wide fitness assay. **(A)** Determination of IC-50 for drimenol (1) activity against *C. albicans* and *S. cerevisiae*. Yeast growth conditions (YPD medium at 30°C) were used to determine the IC-50 values. The red line in each panel indicates growth of the DMSO reference. An IC-50 of ∼25 μg/ml for *C. albicans* and ∼15 μg/ml for *S. cerevisiae* was calculated for **1**. Albicanol (**2**) showed weaker activity against both fungi and was not considered for further analysis. **(B, C)** Genome-wide screens of *S. serevisiae* (B) and *C. albicans* (C) mutant libraries against drimenol (1) for drug induced hypersensitivity. Pooled collections of *S. cerevisiae* nonessential homozygous and essential heterozygous mutants were grown in the presence and absence of **1** at the concentration of 0.025 mg/ml for the indicated number of generations before profiling for their abundance (DNA barcodes). 20 generations for essential heterozygous and five generations for non-essential homozygous mutants were used. Similarly, *C. albicans* Tn-insertion mutants (heterozygous, 20 generations) were treated with 0.025 mg/ml of **1.** Each spot represents a single mutant. The log ratio of each mutant (**1** exposed vs no drug control) was calculated and presented in scatter plots where greater the number the more sensitive that strain is to the treatment.

In this study, we used *S. cerevisiae* barcoded homozygous nonessential and heterozygous essential, and *C. albicans* barcoded heterozygous Tn mutant [37] libraries. Briefly, IC-50 of **1** for *C. albicans* and *S. cerevisiae* was determined under yeast growth (YPD) conditions (**Figure 4A**). Based on this assay results, IC-50 of 25 μg/ml for *C. albicans* and 15 μg/ml for *S. cerevisiae* was calculated for **1**. Two different sub-MIC concentrations of **1** were selected for determining the mechanism of action against mutant libraries.

Next, pooled *S. cerevisiae* and *C. albicans* mutant collections were grown separately in the presence or absence of compounds (with DMSO) for 20 generations, barcodes from genomic DNA were amplified and relative strain abundances were quantified based on TAG microarray signals. The log_2_ ratio of tag signals between DMSO control and **1** exposed samples is presented in scatter plots as the “fitness defect” (**Figures 4B** and **C**). Mutants that were depleted from the growth pool due to **1** are indicated by circles. Mutants that were highly susceptible to **1** are shown with high log ratios (e.g. SEC66 in **Figure 4B**; highly depleted in the pool) and considered putative targets. Lists of *S. cerevisiae* and *C. albicans* mutants that are highly sensitive to **1** are listed in Supplementary Tables 1 and **2**, respectively.

**Figure 5:**
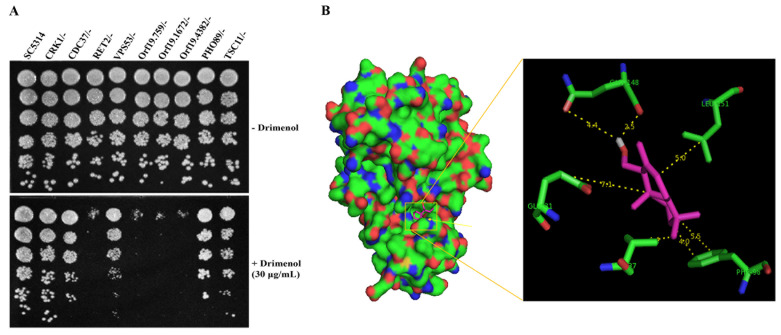
FIGURE 5: Yeast spot assay and molecular docking. **(A)** Validation of *C. albicans* Tn-insertion mutant screen data by yeast spot assay. A four-fold serially diluted yeast cultures of indicated *C. albicans* heterozygous mutants (GRACE) were spot tested on YPD agar containing **1** (30 μg/ml) or DMSO (− **1**). Heterozygous mutants (*RET2/-, Orf19.759/-, Orf19.1672/-*, and *Orf19.4382/-*) directly or indirectly affected by **1** were hypersensitive and showed lack of growth. **(B)** Molecular docking of drimenol with the *C. parvum* Crk1 kinase. Molecular docking of **1** with *C. parvum* Crk1 crystal structure (2QKR-A) was performed using AutoDock Vina software.

Our results of forward genetic screening from mutant libraries of *C. albicans* and *S. cerevisiae* with **1** indicate that it affects cellular activities involved in protein secretion, vacuolar functions, chromatin remodeling and cyclin dependent protein kinase (CDK)-associated functions (**Figure 5A** and Supplementary Tables 1 and 2). For example, *SEC66*, highly sensitive to **1** (log_2_ >4.5, **Figure 4B**), is a component of Sec63 SECretary complex in *S. cerevisiae*, involved in protein targeting and import into the ER. Similarly, *VMA22* is a vacuolar membrane ATPase required for vacuolar H+-ATPase function and localized to the yeast ER (*Saccharomyces* Genome Database).

The *ISN1* (log_2_ 4.4, **Figure 4C**) gene product is involved in inosine 5'-monophosphate 5'-nucleotidase activity in *C. albicans* (*Candida* Genome Database). This gene product is uncharacterized and it is present only in fungi and not in human or murine cells, suggesting that Isn1p a suitable antifungal drug target. *IFG3* is a putative D-amino acid oxidase, which is uncharacterized, and *CRK1* is a protein kinase of the Cdc2 subfamily involved in hyphal development and virulence in *C. albicans* [38]. The *CRK1* ortholog in *S. cerevisiae* is *SGV1,* which is a part of the BUR2 kinase complex and plays a major role in transcriptional regulation.

### Yeasts spot assay to validate drimenol (1) mechanism of action

Based on the forward genetic library screening assay results (**Figure 4**) and the functions of putative targets inferred from the available literature, we selected a few heterozygous mutants of *C. albicans* that had high to medium positive log_2_ ratios (hypersensitive, *CRK1* and its putative interacting partners proteins *CDC37, Orf19.759, Orf19.1672 and Orf19.4382*) and a few with negative log_2_ ratios (resistance, *VPS53, TSC11* & *PHO89*) to verify the genetic screening data. Agar medium containing a sub-MIC concentration of **1** (30 μg/ml) or solvent was used to spot test suspensions of various mutants (GRACE mutant collection) [39].

Results shown in **Figure 5A** confirm the findings of the forward genetic screening assays of *C. albicans* mutants. For example, Crk1 Kinase is predicted to associate with *RET2*, which is involved in retrograde vesicle transport for protein signaling and secretion (*SEC*66), and plays a role in protein translation with G1/S cell-cycle transition [40]. Products encoded by Ret2, orf19.759 (*SEC21*), orf19.1672 (*COP*1) and orf19.4382 (*RET3*) are uncharacterized and are likely targets of Crk1 kinase, which are defective in growth on agar medium containing **1** (**Figure 5A**). Molecule **1** induced hypersensitivity of these mutants, which represent candidate targets/pathways. The orf19.759 (*SEC21* ortholog of *S. cerevisiae*) is uncharacterized in *C. albicans*. Sec21 is involved in the anterograde transport of vesicles from the endoplasmic reticulum (ER) to the Golgi, in the retrograde transport from the Golgi to the ER, COPI vesicle coat, and endosome localization [41]. *RET2* is also uncharacterized in *C. albicans* and the ortholog is a subunit of the coatomer complex (COPI), which coats Golgi-derived transport vesicles, is involved in retrograde transport between the Golgi and the ER, and interacts with Crk1 kinase in the two-hybrid system [42]. Crk1 is known to play a role in regulating trafficking and secretion of effectors by interacting with the early endosome during *Ustilago maydis* (corn smut fungus) infection in corn plants [43, 44].

Since Crk1 kinase may interact with multiple targets (Ret2, orf19.759, orf19.1672 and orf19.4382) and because Crk1 represents an important antifungal drug target [38], we performed computational molecular docking of **1** using the *Cryptosporidium parvum* Crk1 crystal structure (2QKR-A) [45] and the AutoDock Vina software [46]. Results shown in **Figure 5B**, suggest that **1** interacts with the N-terminal catalytic domain of *C. albicans* Crk1 (which has 61% similarity and 40% identity to the *C. parvum* Crk1). Particularly noteworthy from our computational docking studies is that **1** shows close interactions with Gly 31, Val 37, Gln 148, Leu 151, and Phe 98 amino acid residues. The docked structure shows that an available open space in Crk1 for incorporation of an additional functional group to the cyclohexane A ring of **1** (**Figure 1**), signifying a possible modification of **1** for future improvement of biological activity. Thus, this CDK member may comprise the target of **1;** of note, this conserved gene is present in many of the tested pathogenic fungi (**Table 2**).

## DISCUSSION

In our search for novel antifungal small molecules from our available synthetic terpenoids, we have identified two compounds, (-)-drimenol (**1**) and (+)-albicanol (**2**) (**Figure 1**), that show strong activity against *C. albicans*. Among these two compounds, **1** shows stronger bioactivity. It acts not only against *C. albicans* in a fungicidal manner but also against *A. nidulans*, FLU resistant strains of *C. albicans, C. glabrata, C. krusei, Cryptococcus* spp. and dermatophytes, suggesting that **1** is a broad-spectrum antifungal agent (Supplemenatary Figures 1, 2 and **Table 2**). At an increased concentration (100 μg/ml), **1** causes rupturing of the fungal cell wall/membrane, e.g. in *C. albicans* (Supplemenatary Figure 2) and *Cryptococcus* spp. (data not included). *C. auris* is an emerging and multidrug resistant strain that causes nosocomial infections and has been reported recently across the world [33]. Our bioscreen-based growth curve monitoring assay with **1** showed better activity than the clinically used antifungal drug FLU (**Figure 2**) indicating a potential use of **1** against *C. auris* and other drug-resistant fungal pathogens. Since molecule **1** is effective against the antifungal resistant *C. auris, C. albicans* and certain strains of *C. neoformans* (**Table 2**) and the MOA of **1** is different from FLU or other clinical antifungals, molecule **1** can be a useful additional antifungal agent with novel target(s). Molecule **1** synergizes FLU activity (FICI <0.5) in a checkerboard assay against *C. albicans* (data not shown) suggesting its potential utility in combinatorial antifungal therapy.

To understand the broad-spectrum antifungal potential, we evaluated **1** against various fungi that are pathogenic to humans and determined its mechanisms of action in *C. albicans* and *S. cerevisiae*. Based on our yeast mutant screening data and subsequent spot assay results, we found that **1** acts as a fungicidal compound by affecting cellular activities targeting protein trafficking between Golgi and ER, protein secretion (Sec system) and cell signaling, possibly through cell division related kinase 1, Crk1 (**Figures 4B** and **C** and **5A**). Genetic methods have been used to determine the mechanism of antifungal compounds by drug-induced hypersensitivity assays [36, 47]. Using similar approaches, we showed that **1**-mediated inhibition of *C. albicans* heterozygous mutants of *CDC37, Orf19.759, Orf19.1672* and *Orf19.4382*, the known or putative targets of Crk1 kinase, at sub-MIC concentration. Ac-cording to Nelson [48], the Cdc2 kinase (Crk1 kinase is a member of Cdc2 subfamily) plays a major role in regulating the retrograde membrane flow from the Golgi to the ER either alone or with another kinase kinase (e.g. MEK1) during mitosis. We speculate that molecule **1** may likely be disrupting the interaction of Crk1 with one or more of these gene products. In support of this observation, computational molecular docking of **1** with the crystal structure of a fungal (*C. parvum*) Crk1 kinase showed interactions of **1** with the key residues in the catalytic domain (N-terminal) of Crk1 (**Figure 5B**).

Molecule **1**, a natural product, is present in liverworts and higher plants [49–52] and its antifungal activities against various pathogenic fungi have been reported previously though with higher MIC values. A recent study found that **1** had antifungal activity against *Botrytis cinerea*, a plant fungal pathogen, and the mechanisms appear to involve fungal membrane damage and reactive oxygen species (ROS) production in the germinating spores [52]. From our forward genetic screening and yeast spot assays, we identified Ret2 and related other gene products (orf19.759, orf19.1672, and orf19.4382) as hypersensitive to **1**. Since Crk1 interacts with these SEC/COPI complexes [42–44, 53] that traffic protein cargo between ER and Golgi, we surmise that molecule **1** by targeting these complexes may cause ROS production via ER-mediated unfolded protein response (UPR) or other pathways [54]. According to Robles-Kelly *et al.* [52], the ROS production in germinating spores due to drimenol treatment at 80 µg/ml was increased about 1-fold. However, we did not find the modulation of stress response (osmotic/oxidative) genes/pathways from our genetic screen data (**Figure 4B** and **C**, Supplementary Tables 1 and 2). The other possibility could be that physiological responses may vary between fungi (plant vs human pathogen), their growth conditions (media, spores vs yeast cells), and the concentrations of the drug used (20-25 µg/ml for yeasts vs 80 µg/ml for *B. cinerea*) [52]. Further studies are required to determine how molecule **1** affects these pathways.

Cinnamodial is a closely related compound belonging to the drimane sesquiterpenoid family with a potent antifungal activity [55], but its chemical structure (containing two aldehyde groups; **Figure 1**) and physiological properties are quite different from **1**. For example, the antifungal activity of cinnamodial was shown to be abolished by amine compounds (likely due to a coupling reaction from the aldehyde functions of cinnamodial with the amino group of amine compounds) or when cinnamodial was used in YPD medium [4, 56]. In contrast, molecule **1**'s bioactivity was not affected by amines or YPD medium (**Figure 4A**). Thus, the antifungal mechanisms of **1** could be different from cinnamodial. Since the synthetic route for **1** and its analogs are well established, improvements of its antifungal properties are possible through medicinal chemistry approaches and computational docking experiments.

In summary, we have synthesized a focused library of drimane sesquiterpenoid compounds and identified **1** as a broad-spectrum fungicidal compound against various human pathogenic fungi including *C. albicans, C. auris, C. neoformans, Aspergillus, Blastomyces, Pneumocystis*, and dermatophytes at concentrations of 8 - 64 µg/ml. By employing the libraries of bar-coded *C. albicans* and *S. cerevisiae* genome-wide mutants, the MOA of **1** was determined. Further evaluation of **1** in animal models of fungal diseases will help developing **1** as an antifungal agent.

## MATERIALS AND METHODS

### Synthesis of drimane molecules

*(1R,2R,4aS,8aS)-2-Hydroxy-2,5,5,8a-tetramethyl-decahydro-naphthalene-1-carbaldehyde* ***(4)***

0.19 g (0.81 mmol) potassium periodate were added to a solution of 0.20 g (0.74 mmol) triol **3** [27] in 10 ml THF and 2.5 ml water. The resulting mixture was stirred at 25°C for 4 hours, diluted with water (50 ml) and extracted three times with ethyl acetate (50 ml each). The combined extracts were washed with water and brine, dried (anhydrous Na_2_SO_4_), concentrated, and column chromatographed on silica gel using a mixture of hexane and ethyl acetate (20:1) as an eluent, yielding 0.16 g (91% yield) compound **4**, whose spectral data is in agreement with that reported [27].

*(1S,2R,4aS,8aS)-1-(Hydroxymethyl)-2,5,5,8a-tetramethyl-deca-hydronaphthalen-2-ol* ***(5)***

To a cold (0°C) solution of 1.0 g (4.2 mmol) aldehyde **4** in 80 ml of diethyl ether under argon, 80 mg (2.1 mmol) lithium aluminum hydride were added in portions. The resulting solution was stirred at 0°C for 30 minutes, diluted with aqueous NH_4_Cl, and extracted with diethyl ether three times (50 ml each). The combined extracts were washed with water and brine, dried (MgSO_4_), and concentrated, yielding 0.98 g (97% yield) diol **5**, whose spectral data are in agreement with that reported [27].

*[(1S,2R,4aS,8aS)-2-Hydroxy-2,5,5,8a-tetramethyl-decahydro-naphthalen-1-yl]methyl acetate* ***(6)***

To a cold (0°C) solution of 0.10 g (0.40 mmol) diol **5** in 2 ml dichloromethane and 0.32 g (4.0 mmol) pyridine under argon, 49 µl (0.48 mmol) of acetic anhydride were added, and the resulting solution was stirred at 0°C for 30 minutes and 25°C for 1 h. It was diluted with 30 ml of aqueous NH_4_OH, extracted twice with diethyl ether (30 ml each), and the combined extracts were washed with water and brine, dried (anhydrous Na_2_SO_4_), concentrated, and column chromatographed on silica gel using a gradient mixture of hexane and diethyl ether as eluents, yielding 90 mg (80% yield) of acetate **6**. Mp. 64 – 67 °C; [α]^D^_22_ = −8.2 (c = 0.55, CHCl_3_); ^1^H NMR (CDCl_3_; 400 MHz) α 4.35 (dd, *J* = 12, 4 Hz, 1 H), 4.24 (dd, *J* = 12, 4 Hz, 1 H), 2.05 (s, 3 H), 1.88 (dt, *J* = 12, 2 Hz, 1 H), 1.70 – 0.93 (a series of m, 11 H), 1.17 (s, 3 H), 0.88 (s, 3 H), 0.86 (s, 3 H), 0.80 (s, 3 H) ppm; ^13^C NMR (CDCl_3_; 100 MHz) α 171.4, 72.6, 62.6, 60.0, 55.7, 44.0, 41.7, 39.7, 38.1, 33.5, 33.2, 24.6, 21.6, 21.3, 20.3, 18.4, 15.8 ppm. MS (electrospray ionization), m/z 283.1 (M+H^+^). HRMS-ESI: m/z [M + H]^+^ calcd for C_17_H_31_O_3_^+^: 283.2268, found: 283.2273.

*(1S,2R,4aS,8aS)-1-(Hydroxymethyl)-2,5,5,8a-tetramethyl-deca-hydronaphthalen-2-yl acetate* ***(7)***

Compound **7** was prepared by a sequence of three reactions: (i) silylation of the primary alcohol function of **5** with *t*-butyldimethylsilyl chloride; (ii) acetylation of the tertiary alcohol function with acetyl chloride and pyridine; and (iii) removal of the *t*-butyldimethylsilyl ether protecting group with tetra-*n*-butylammonium fluoride in THF.

To a solution of 9.5 mg (40 µmol) compound **5**, 11 mg (150 µmol) imidazole, and 6 mg (49 µmol) 4-(dimethyl-amino)pyridine in 2 ml of dichloromethane under argon at 25°C, 14.3 mg (95 µmol) *t*-butyldimethylsilyl chloride were added, and the solution was stirred for 4 h. The reaction mixture was diluted with 10 ml of aqueous ammonium chloride and extracted with diethyl ether three times (10 ml each). The combined extracts were washed with water (10 ml) and brine (10 ml), dried (anhydrous Na_2_SO_4_), and concentrated, yielding 12.5 mg the mono-silylated product. This crude product was used in the subsequent step without purification. To a solution of the above mono-silylated product and 0.1 ml pyridine in 0.5 ml of dichloromethane under argon at 0°C, 10 µl (0.13 mmol) acetyl chloride were added. The reaction mixture was stirred at 25°C for 2 h, diluted with aqueous ammonium chloride (10 ml), and extracted three times with diethyl ether (10 ml each). The combined extracts were washed with brine, dried (anhydrous Na_2_SO_4_), and concentrated, getting the crude product, which was used in the following step without purification. The above crude product was dissolved in 1 ml of dried THF (distilled over sodium/benzophenone) and 0.3 ml (0.3 mmol) of tetra-*n*-butylammonium fluoride (1 M solution in THF) and stirred at 25°C under argon for 1 h. The reaction solution was diluted with 0.1 N ammonium hydroxide (10 ml) and extracted with diethyl ether three times (10 ml each). The combined extracts were washed with water (10 ml) and brine (10 ml), dried (anhydrous Na_2_SO_4_), concentrated, and column chromatographed on silica gel using a gradient mixture of hexane and diethyl ether, yielding 4.2 mg (38% overall yield from diol **5**) of compound **7**. Compound **7**: Mp. 101 – 103 °C; [α]^D^_22_ = +0.35 (c = 0.23, CHCl_3_); ^1^H NMR (CDCl_3_; 400 MHz) α 3.91 (dd, *J* = 12, 2 Hz, 1 H), 3.84 (dd, *J* = 12, 2 Hz, 1 H), 2.95 – 2.90 (m, 1 H), 1.98 (s, 3 H), 1.88 (dt, *J* = 12, 2 Hz, 1 H), 1.62 (s, 3 H), 1.70 – 0.88 (a series of m, 10 H), 0.94 (s, 3 H), 0.87 (s, 3 H), 0.82 (s, 3 H) ppm; ^13^C NMR (CDCl_3_; 100 MHz) α 169.9, 84.9, 63.8, 59.8, 55.8, 41.8, 39.5, 38.2, 36.1, 33.5, 33.2, 25.8, 22.8, 21.7, 18.3 (2 C), 16.1 ppm. MS (electrospray ionization), m/z 305.1 (M+Na^+^). HRMS-ESI: m/z [M + Na]^+^ calcd for C_17_H_30_NaO_3_^+^: 305.2087, found: 305.2082.

*(1S,2R,4aS,8aS)-1-(2,2-Dimethyl-1,3-dioxolan-4-yl)-2,5,5,8a-tetramethyl-decahydronaphthalen-2-ol* ***(8)***

A solution of 18 mg (67 µmol) triol **3**, 50 µl 2,2-dimethoxy-propane and 3 mg of anhydrous *p*-toluenesulfonic acid in 1 ml of toluene was stirred under argon at 55°C for 1 h. The solution was cooled to room temperature, neutralized with sodium bicarbonate (∼3 mg), diluted with 10 ml of water, and extracted with ethyl acetate three times (15 ml each). The combined extracts were washed with brine, dried (MgSO_4_), concentrated and column chromatographed on silica gel using a gradient mixture of hexane and diethyl ether as eluent, yielding 14 mg (71% yield) compound **8** as a mixture of two stereoisomers: (the major isomer was partially purified and reported) Mp. 114 – 117 °C; [α]^D^_22_ = −25.1 (c = 1.0, CHCl_3_); ^1^H NMR (CDCl_3_; 400 MHz) α 4.96 (s, 1 H, OH), 4.24 – 4.20 (m, 2 H), 3.59 (td, *J* = 8, 4 Hz, 1 H), 1.84 (dt, *J* = 12, 2 Hz, 1 H), 1.70 – 0.83 (a series of m, 11 H), 1.45 (s, 3 H), 1.41 (s, 6 H, 2 CH_3_), 0.97 (s, 3 H), 0.90 (s, 3 H), 0.83 (s, 3 H) ppm; ^13^C NMR (CDCl_3_; 100 MHz) α 107.4, 73.5, 72.8, 62.2, 55.7, 42.8, 41.5, 40.4, 37.3, 33.6, 33.3, 26.5, 26.2, 25.8, 21.7, 19.7, 18.4 (2 C), 16.1 ppm. MS (electrospray ionization), m/z 333.1 (M+Na^+^). HRMS-ESI: m/z [M + Na]^+^ calcd for C_19_H_34_NaO_3_^+^: 333.2406, found: 333.2411.

### Determination of the antifungal activity of the synthetic compounds

Synthetic pure drimenol or albicanol was dissolved in DMSO (10 mg/ml as stock solution) and used for determining their antifungal activities (minimum inhibitory concentration, MIC) against various fungi according to the microdilution assay of CLSI [32]. The CLSI broth dilution methods M27-A3 for yeasts and M38-A for filamentous fungi were used to determine the susceptibility. Since our initial assay with *C. albicans* confirmed the antifungal activity of drimenol and albicanol, we extended the susceptibility assay to other pathogenic fungi including FLU resistant *C. albicans*, various species of *Candida, Cryptococcus, Aspergillus* and a dermatophyte fungus [57] (strains were generously provided by Dr. Ted C. White at The University of Missouri Kansas City (UMKC). *C. auris* [58] was obtained from Dr. Baha Abdalhamid at The University of Nebraska Medical Center, Omaha NE). Briefly, yeast cells or conidia (for filamentous fungi) were suspended in RPMI-1640 medium to a final concentration of 10^5^ cfu/ml and distributed in 96-well microplates to a total volume of 100 μl/well. Drimenol or albicanol was added into the wells and a two-fold serial dilution was made. Duplicates were used for each concentration and wells with or without DMSO served as controls. Plates were incubated without shaking at 37°C for 24 - 48h for yeasts and 30°C for four days for filamentous fungi (*Aspergillus* spp. and *Trichophyton* spp.). The MIC was defined as the lowest compound concentration at which no growth occurred, as determined visually and microscopically (inverted microscope).

### Determination of *C. auris* growth inhibition by drimenol

The effect of drimenol on the growth of *C. auris* was determined by the Bioscreen-C real time growth monitoring system (Oy Growth Curves Ab Ltd, Finland) as described earlier [59]. The antifungal resistant *C. auris* strain [58] (South Asian Clade) [60, 61] was used. Briefly, 200 µl of RPMI medium containing exponentially growing *C. auris* yeast cells (each at 0.07 OD_600_) were added into the honeycomb wells with or without compound (control) and growth rates were measured for 20 hours at 37°C. Compound treatment was done with two different concentrations for drimenol (50 and 60 µg/ml). The absorbance was measured at 600 nm in 30 min intervals for 24 h at 37°C with shaking for 10 s before each read. A solvent negative control (DMSO) and a FLU (60 µg/ml; antifungal drug) positive control were included in the study. The experiments were repeated at least two times with three technical replicates.

### *Caenorhabditis elegans* (nematode) host model for the *C. albicans* infection assay

The nematode model of candidiasis was used as described before [34] to determine the antifungal efficacy of **(1)**. Briefly, larvae (L2) were fed on a *C. albicans* yeast lawn on YPD agar plate. After collecting the larvae and washing off yeast cells with PBS buffer, an aliquot of larvae was mixed with buffered RPMI medium and distributed to microtiter wells. Drimenol **(1)** (50 μg/ml) or DMSO (solvent control) were added to microtiter wells equally containing the worms. As a positive antifungal control, amphotericin B (1 μg/ml) was included. Triplicate wells, about 20-30 nematodes/well, were used. The assay plate was placed in a plastic box lined with moisture paper and incubated at 30°C for 2-3 days. Nematodes were monitored under an inverted microscope and recorded with a digital camera connected to a microscope.

### Yeast spot assay

Yeast Peptone Dextrose (YPD) agar containing a sub-MIC concentration of drimenol (30 µg/ml) or an equal volume of DMSO was used to spot test the *C. albicans* heterozygous mutants (GRACE library [39]). Yeast suspensions of various mutants and the wild type *C. albicans* were used. 5 μl of a four-fold serially diluted suspension were spotted on the agar plates and incubated at 30°C for yeast growth for 24 h, and photographed. Experiments were repeated at least three times and a representative result was shown.

### Genome-wide fitness assay

The *Saccharomyces* yeast deletion collection was comprised of approximately 5,900 individually bar-coded heterozygous diploid strains (HIP [haploinsufficiency profiling]) and ∼4,800 homozygous diploid strains (HOP [homozygous deletion profiling]). Pools of approximately equal strain abundance were generated by robotically pinning (S and P Robotics, Ontario, Canada) each strain (from frozen stocks) onto YPD agar plates as arrays of 384 strains/plate [35, 62, 63]. After two days of growth at 30°C, colonies were collected from plates by flooding with YPD, and cells were adjusted to an optical density at 600 nm (OD_600_) of 2. The fitness of each strain in each experimental pool was assessed as described previously [35]. The dose that resulted in 15% growth inhibition in *S*. *cerevisiae* BY4743 (the parent strain of the yeast deletion collection) was determined by analyzing dose response over the course of 16 h of growth at 30°C. Screens of the homozygous deletion collection were performed over five generations of growth and screens of the heterozygous deletion collection were collected after 20 generations of growth. Cells were processed as described previously [35]. Genomic DNA was extracted from each sample and subjected to PCR to amplify the unique bar code identifiers. The abundance of each bar code was determined by quantifying the microarray signal as previously described [35]. *C. albicans* pooled screens used the tn-transposon collection [37]. Growth assays were performed in duplicates and samples were recovered after 20 generations of growth. Genomic DNA extraction, tag amplification, and hybridization were performed as described above.

## SUPPLEMENTAL MATERIAL

Click here for supplemental data file.

Click here for supplemental data file.

Click here for supplemental data file.

All supplemental data for this article are available online at http://www.microbialcell.com/researcharticles/2020a-edouarzin-microbial-cell/.

## Abbreviations:

CLSI: – clinical laboratory standard institute,
DMSO: – dimethyl sulfoxide,
ER: – endoplasmic reticulum,
FLU: – fluconazole,
MOA: – mechanism of action,
ROS: – reactive oxygen species.
