# Supplementary material for: Broad-spectrum antifungal activities and mechanism of drimane sesquiterpenoids
Source: Microb Cell. 2020 Mar 12;7(6):146–59. doi: 10.15698/mic2020.06.719 (PMC7278516; doi:10.15698/mic2020.06.719)
Supplement: Supplementary file 1 [file mic-07-146-s01.pdf]

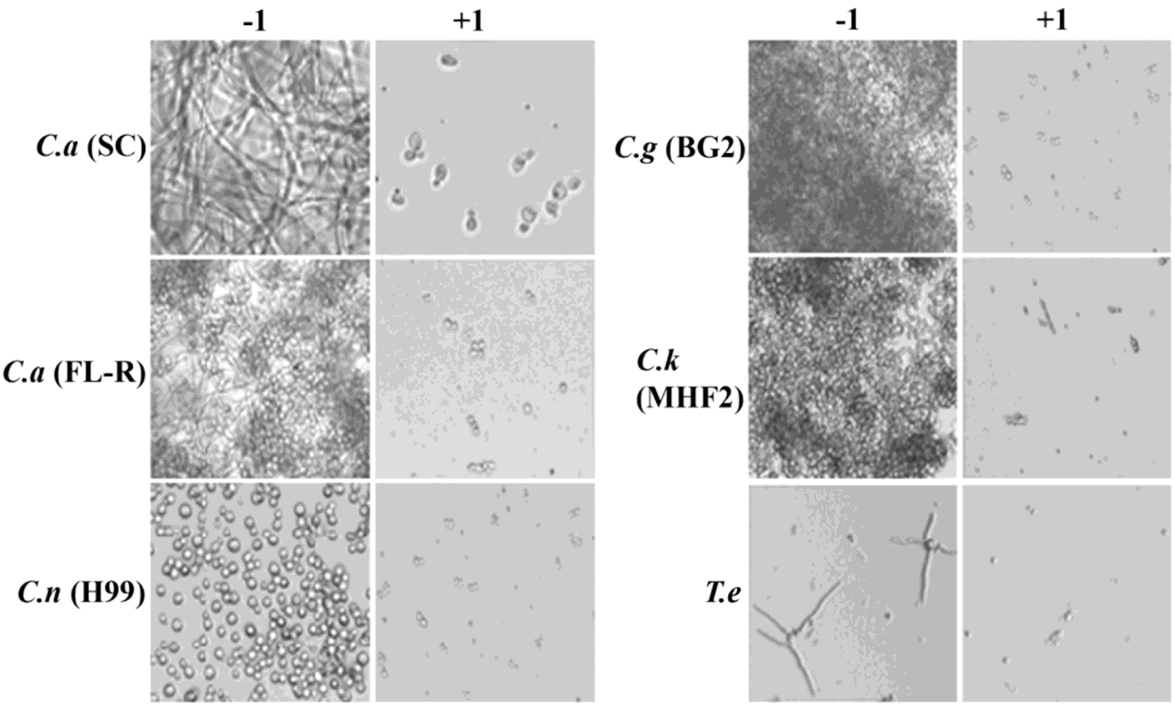

SUPPLEMENTARY FIGURE S1: Antifungal activity of 1 against various human pathogenic fungi. The CLSI broth dilution methods of M27-A3 for yeasts and M38-A2 for filamentous fungi were used. Drimenol (**1**) was dissolved in DMSO and a two fold serial dilution was used between 200 - 12.5 µg/ml. Representative microscopic images (Leica, inverted microscope) taken at 200X magnification from **1** with cells exposed 50 µg/ml (MIC), except *T. equinum* which was exposed to 15 µg/ml at 30 ^{o}C, are shown. The MIC for **1** is 50 µg/ml under these assay conditions except mentioned otherwise. *C. a* (SC), *Candida albicans* (SC5314); *C. a* (FL-R), *C. albicans* FLU-resistant; *C. g* (BG2), *C. glabrata* (BG2); *C. k.* (MHF2), *C. krusei* (MHF2); *C. n* (H99), *Cryptococcus neoformans* (H99); *T. e, Trichophyton equinum*.

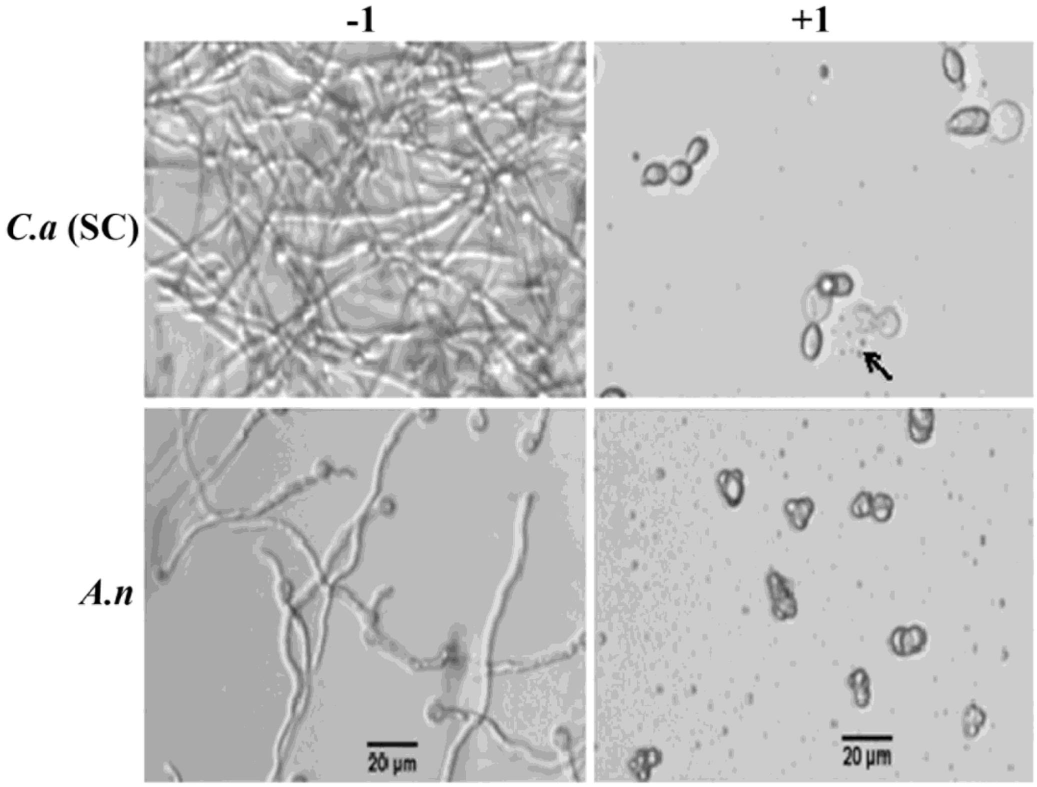

SUPPLEMENTARY FIGURE S2: Antifungal activity of 1 against *C. albicans* and *A. nidulans*. The CLSI broth dilution methods of M27-A3 for yeasts and M38-A2 for filamentous fungi were used. *C. albicans* (SC5314) showed lysis of yeast cells (arrow) at 100 µg/ml of **1** compared to the control where a network of hyphal growth was observed**.** Similarly, the germination of *Aspergillus nidulans* spores was inhibited by **1** (lower, right panel). Representative microscopic images (Leica, inverted microscope) were shown. Scale bar, 20 µm.

SUPPLEMENTARY TABLE S1. *S. cerevisiae* genetic screening data.
Supplementary Table S1 is attached as an excel file.

SUPPLEMENTARY TABLE S2. *C. albicans* genetic screening data.
Supplementary Table S2 is attached as an excel file.
